# Splenic Rupture in Infectious Mononucleosis: A Case Report

**DOI:** 10.7759/cureus.83438

**Published:** 2025-05-03

**Authors:** Ahmer A Longi, Zubair Edakkavil, Misbah Fazlani, Nambiar Rajesh, Mohammed O Quraishi

**Affiliations:** 1 Department of Internal Medicine, Mediclinic Welcare Hospital, Dubai, ARE; 2 Department of Surgery, Mediclinic Welcare Hospital, Dubai, ARE

**Keywords:** atraumatic splenic rupture, epstein-barr virus, infectious mononucleosis, splenectomy, splenic rupture

## Abstract

Splenic ruptures are rarely seen in infectious mononucleosis (IM), caused by the Epstein-Barr virus (EBV). It is a self-limiting viral infection characterized by fever, pharyngitis, and lymphadenopathy. While most cases are mild and resolve without complications, rare and severe complications can arise, including splenic rupture, which is a potentially life-threatening event. Previous case reports have linked splenic rupture to IM with increased splenic size. We present an atypical case report of splenic rupture in a young adult diagnosed with IM, whose spleen was found to be of normal size. This study emphasizes the need for physicians to be more vigilant and closely observe for splenic ruptures in IM patients, despite having normal splenic sizes. This would contribute to reducing both the mortality and morbidity associated with atraumatic splenic ruptures.

## Introduction

Infectious mononucleosis (IM) is a common infection caused by Epstein-Barr virus (EBV) [1]. EBV is a member of the gamma herpes virus family. EBV typically causes mild symptoms when contracted in childhood and establishes a lifelong dormant infection. However, if the initial infection occurs during adolescence, EBV results in IM in 35-50% of cases [2]. IM is characterized by fever, pharyngitis, and generalized lymphadenopathy [3]. Other common symptoms include fatigue, malaise, and headaches. Symptoms of IM typically appear after an incubation period of four to seven weeks [4]. Transmission occurs via salivary droplets and requires close contact [5]. IM symptoms typically resolve within three to eight weeks. Splenomegaly is observed in nearly all cases, with splenic size increasing by three to four times that of the baseline [6]. The spleen is a highly vascularized organ that filters about 10-15% of the total blood volume each minute, and its rupture, whether from the parenchyma or splenic blood vessels, can lead to substantial blood loss [7]. Although rare, occurring in fewer than 0.5% of infected individuals, splenic injury or rupture is among the most concerning complications [1]. Most cases of IM are self-limiting and have an excellent prognosis; however, in rare instances, acute complications such as splenic rupture, hepatitis, and significant tonsillar enlargement leading to airway obstruction can occur [4]. Splenic rupture is generally classified into two types: traumatic and atraumatic. A diagnosis of atraumatic splenic rupture (ASR) can be established based on the Orloff and Peskin criteria, which require the following four conditions: (1) a detailed history shows no prior trauma; (2) there is no evidence of disease in other organs that could cause splenic rupture; (3) no peri-splenic adhesions or scarring indicative of trauma or previous rupture are present; and (4) the spleen appears normal on gross histological examination [8]. The causes of ASR can be grouped into seven primary categories: neoplastic, infectious, hematologic, inflammatory, iatrogenic, primary splenic disorders, and idiopathic. Neoplastic and infectious diseases account for over half of the cases [9].

In the case presented below, we report an instance of ASR resulting from an infectious etiology, specifically due to EBV infection. The patient was managed successfully through an emergent splenectomy, which was deemed necessary due to the severity of the rupture and associated hemodynamic instability.

## Case presentation

**Figure 1:**
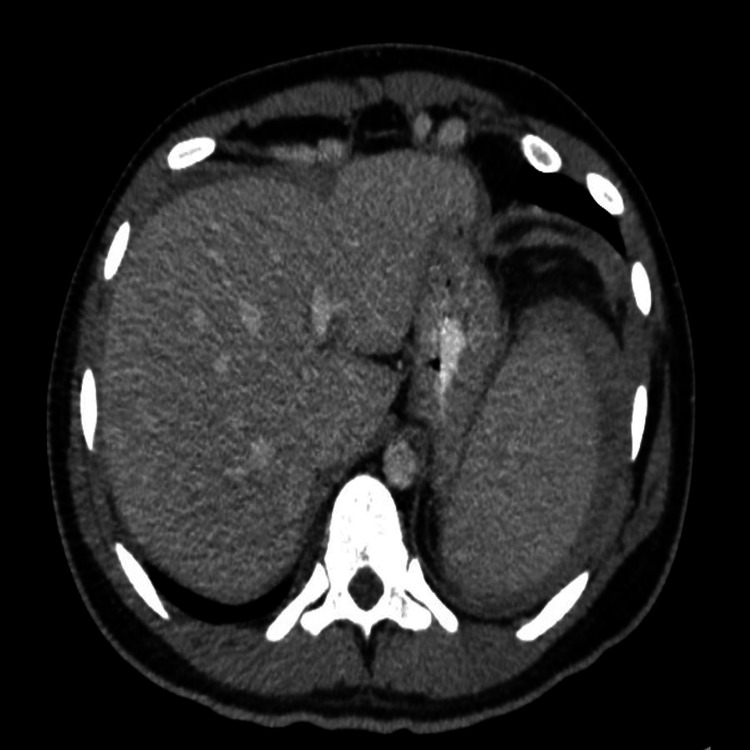
CT scan of the abdomen with intravenous contrast in the transverse plane

**Figure 2:**
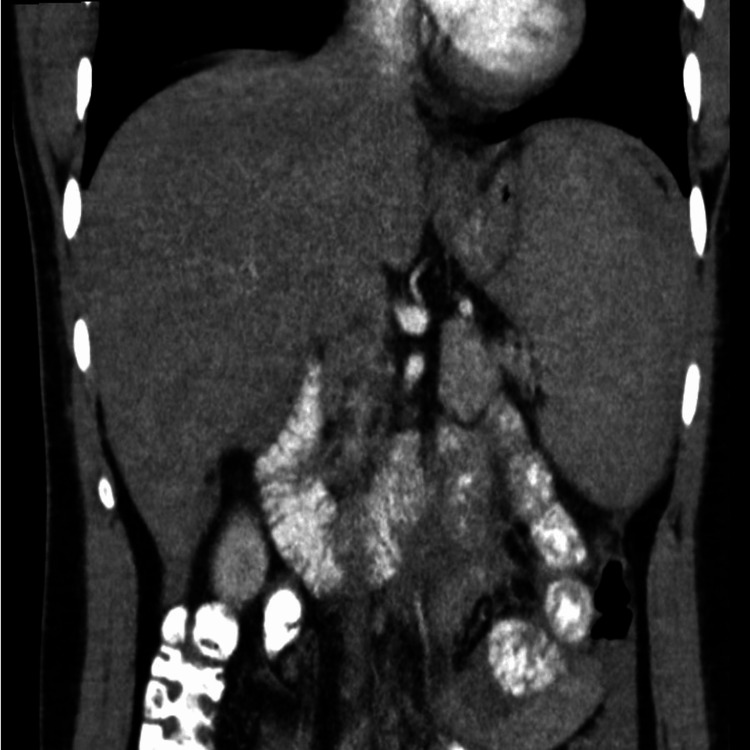
CT scan of abdomen with intravenous contrast in the sagittal plane

A 26-year-old gentleman was admitted to the emergency department with chief complaints of fever, chills, dry non-productive cough, and body aches for one week. One day prior, he developed abdominal pain, which was intermittent and dull, radiating to the left shoulder with a pain scale of 4/10. He had a recent history of iron deficiency anemia and had just finished his oral iron regimen a month before his presentation. The patient did not have high-risk sexual or social factors. Pet cats were fully vaccinated at home. He is a professional engineer who occasionally plays football. Initial vital signs included the following vitals: a blood pressure of 114/64 mmHg, heart rate of 70 beats/min, respiratory rate of 17 breaths/min, SpO_2_ of 99% at room air, and temperature of 37.6°C. On the initial examination, the patient appeared calm and undistressed. Jaundice and regional lymphadenopathies were not observed. The throat exam showed mild tonsillar hyperemia. The examination of the respiratory, cardiovascular, and neurological systems was within the normal limits. The abdomen was soft and tender in the epigastric area and the left upper quadrant, with no signs of hepatosplenomegaly. There was no guarding on palpation, and bowel sounds were normal. Chest radiography revealed prominent bronchovascular markings with no signs of respiratory infection. Electrocardiography (ECG) showed a normal sinus rhythm with a normal axis and no S-T or T-wave abnormalities. Initial blood work included full blood counts as follows: haemoglobin at 15.2 g/dL, white cell count at 4.3 x 10^3^/uL (predominantly lymphocytes), platelet count at 155 x 10^3^/uL; CRP at 32 mg/L, and creatinine at 86.4 umol/L. Liver function tests (LFTs) showed total bilirubin (TB) at 14.70 umol/L, direct bilirubin (DB) at 8.80 umol/L, alkaline phosphatase (AP) at 266 U/L, alanine transaminase (ALT) at 494 U/L, aspartate aminotransferase (AST) at 368 U/L, and albumin at 24.00 g/L. In the electrolytes, sodium was 140 mmol/L, potassium was 3.9 mmol/L, bicarbonate was 19.4 mmol/L, and the rest were within the normal limits. Given the initial presentation, the patient was admitted with an elevated temperature. Blood cultures were sent, and the patient was started on empirical third-generation cephalosporins. Mycoplasma, the influenza rapid test, and strep-A rapid test results were negative. Ultrasonography performed on the same day revealed a fatty liver and mild-to-moderate ascites. This was followed by a computed tomography (CT) scan of the abdomen with contrast due to persistent abdominal pain, which revealed a fatty liver with moderate ascites and no signs of portal hypertension. Liver and spleen sizes were normal (Figures 1-2).

**Figure 3:**
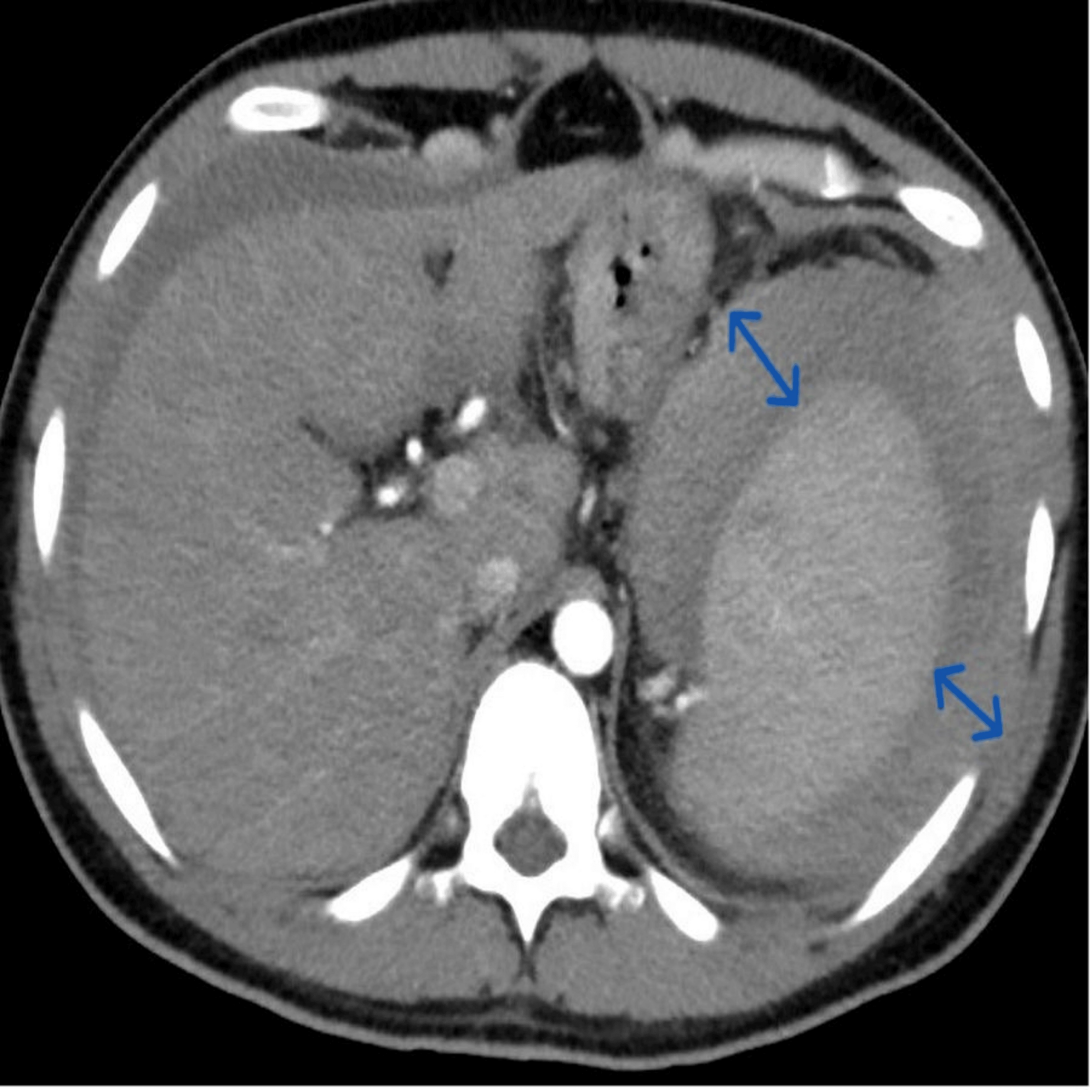
CT angiogram of the abdomen in the transverse plane showing a peri-splenic collection (blue arrows)

**Figure 4:**
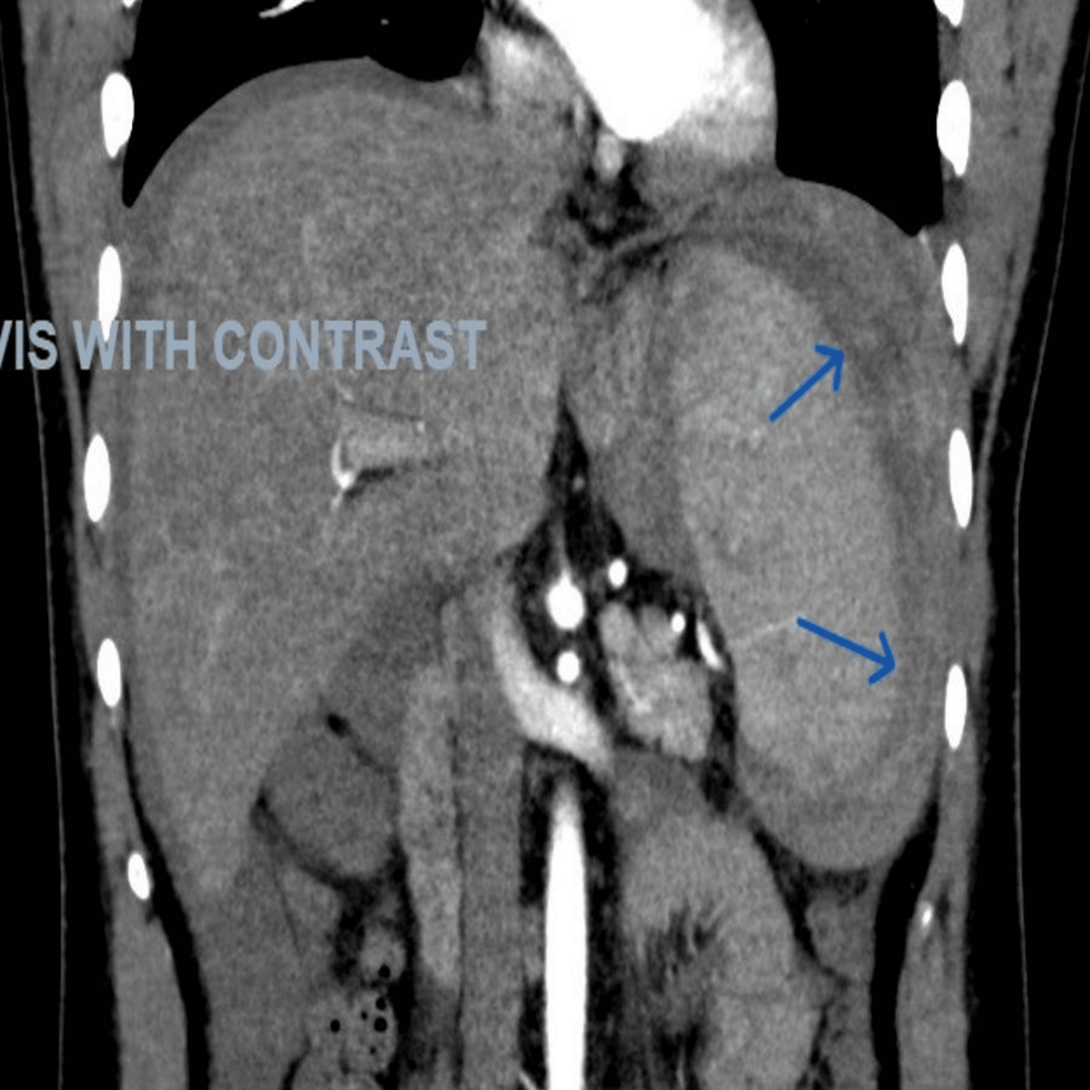
CT angiogram of the abdomen in the sagittal plane showing a peri-splenic collection (blue arrows)

**Figure 5:**
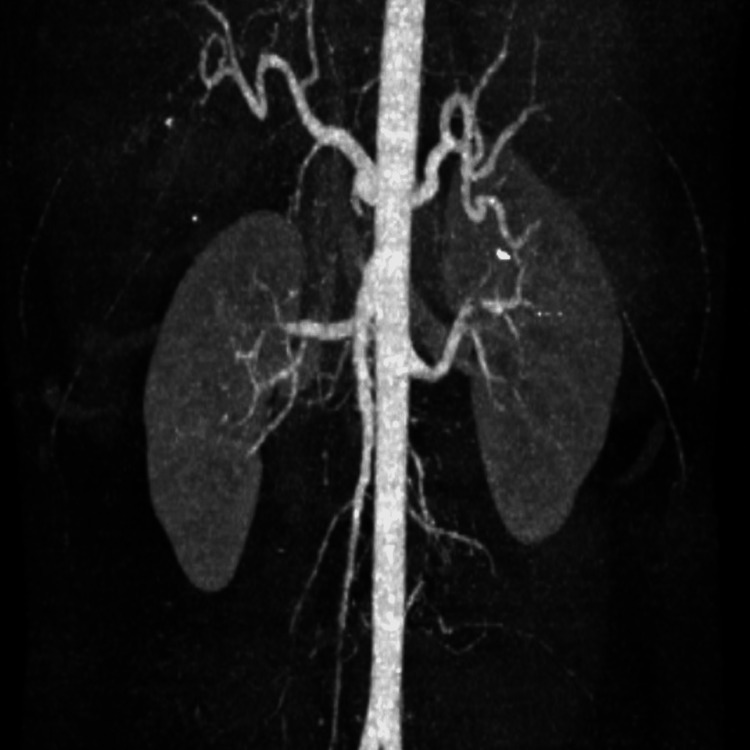
CT angiogram of the abdomen showing absence of a splenic artery pseudoaneurysm

**Figure 6:**
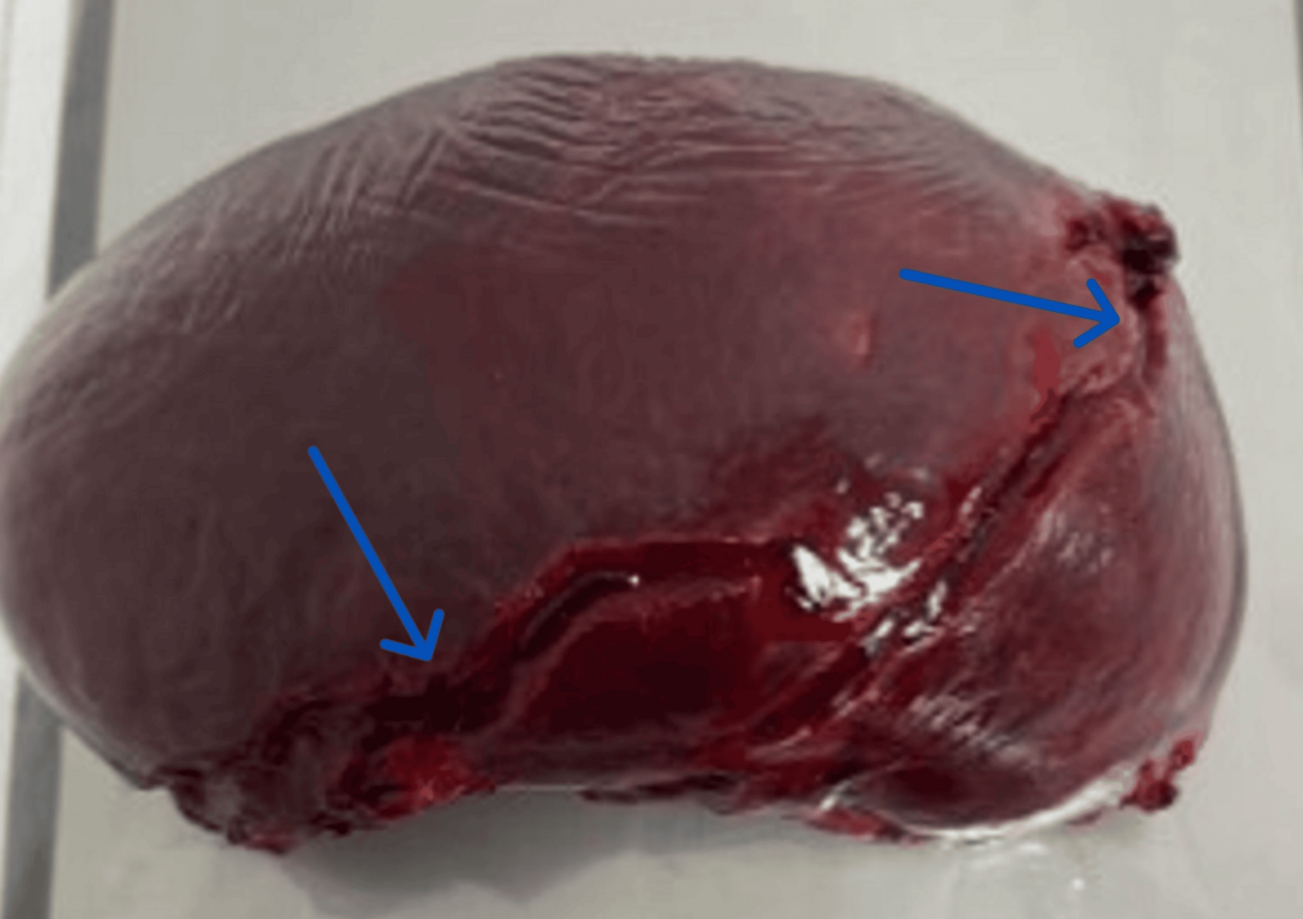
Spleen specimen showing two lacerations (blue arrows)

EBV IgM test results were positive. A diagnostic ascitic tap was performed, which showed hemorrhagic fluid exudate on biochemical examination. The patient’s hemoglobin level dropped to 11.9 with mild abdominal pain. A gastroenterologist, an interventional radiologist, and a general surgeon were also consulted, and a shared decision was made that with any further drop in hemoglobin level, the patient would undergo a CT angiography to determine any source of bleeding. The next day, the patient had further dropped his hemoglobin along with being tachycardic at 126 beats/min, and a CT angiogram was done (Figures 3-5), which showed “features of hyperdense hemorrhagic perisplenic collection. Small non-enhancing hypodensities were noted in both the upper and lower poles of the spleen, suggesting splenic tears.” He underwent emergent splenectomy (Figure 6) and was observed in the intensive care unit (ICU) postoperatively. The intra-abdominal drain was removed, along with bed-to-chair mobilization and incentive spirometry.

The patient’s ICU course was uneventful, as no further decrease in haemoglobin level was observed, and his inflammatory markers and LFTs showed improvement, due to which his care unit was stepped down. The patient continued to undergo reverse isolation in the hospital ward. Meanwhile, he continued to develop a low-grade intermittent fever. However, increased CRP levels and LFT results were observed. Labs included CRP at 118.9 mg/L, TB at 26.6 umol/L, DB at 18.9 umol/L, ALT at 431 U/L, AST at 321 U/L, and AP at 791 U/L. This increase in LFT was unexplained, and an autoimmune workup (antinuclear antibody (ANA) profile, anti-neutrophil cytoplasmic antibodies (ANCA), and liver-kidney microsomal (LKM) antibodies) was also performed (Table 1). A repeat abdominal CT was also performed, which showed post-splenectomy status and no further intra-abdominal collection. Additionally, the antibiotics were escalated to piperacillin-tazobactam. Inflammatory markers and LFT improved. The patient was discharged in stable condition for follow-up in the clinic.

**Table 1:** Laboratory investigations ANA: Anti-nuclear antibody; MPO: Myeloperoxidase; ANCA: Anti-neutrophil cytoplasmic antibody; TTG: Tissue transglutaminase; PR3: Proteinase 3; LKM: Liver-kidney microsomal; VCA: Viral capsid antigen; EBNA: Epstein-Barr nuclear antigen

Labs	Reference range	Results
ANA	Negative	Negative
Faecal calprotectin	Negative: <50 ug/g	24 ug/g
MPO-ANCA	Negative: <20 CU	174 CU
PR3-ANCA	Negative: <20 CU	65.5 CU
Ceruloplasmin	0.2-0.6 g/L	0.39 g/L
Anti-LKM antibody	Negative: <10 U/mL; positive: >10 U/mL	2.0 U/mL
Anti-TTG antibody IgG	Negative: <20 CU	16.8 CU
Anti-TTG antibody IgA	Negative: <20 CU	3.1 CU
Anti-M2 antibody	Negative: <10 IU/mL	1.0 IU/mL
Ascitic fluid cytology	Negative	Negative for malignancy
Anti-VCA IgM	Negative	Positive
Anti-VCA IgG	Negative	Positive
Anti-EBNA IgG	Negative	Negative
Monospot	Negative	Negative

## Discussion

In 1861, Rotinsky first reported an ASR in a leukemic patient, and in 1874, Atkinson first described the rupture of a normal spleen [10]. The spleen is an organ of the reticuloendothelial system used for blood purification. It has a rich collateral blood supply from the splenic and short gastric arteries, owing to the proximity of the organs to the stomach. Nonetheless, it gains 5% of cardiac output [11]. The clinical course of IM is typically characterized by nonspecific symptoms, followed by spontaneous recovery [12].

IM is a prevalent illness that typically presents with the classic triad of lymphadenopathy, fever, and sore throat (pharyngitis). Most cases are self-limiting and managed in primary care with appropriate symptomatic advice. It can be associated with hepatosplenomegaly, jaundice, and temporary derangement of LFTs [13]. EBV is transmitted through salivary secretions. EBV infects the epithelium of the oropharynx and salivary glands and spreads through the bloodstream [14]. EBV can involve virtually any organ system and is linked to a wide range of disease manifestations, including pneumonia, myocarditis, pancreatitis, mesenteric adenitis, myositis, glomerulonephritis, and splenic rupture or infarction [3]. EBV can be diagnosed through a positive heterophile antibody test or by detecting atypical lymphocytes. A definitive diagnosis is confirmed by testing for IgM and IgG antibodies against the viral capsid antigen (VCA) and Epstein-Barr nuclear antigen (EBNA). VCA IgM is a reliable indicator of primary infection and is typically present in most cases when symptoms are evident [5]. The Hoagland criteria, as one diagnostic guideline, require the presence of at least 50% lymphocytes and at least 10% atypical lymphocytes, accompanied by fever, pharyngitis, and adenopathy. A positive serological test is also necessary to confirm the diagnosis [15].

Data from a large medical record database indicate that approximately one in four splenic ruptures occur within the first 21 days following the onset of symptoms [6]. While splenic rupture is most often the result of trauma, it can also occur in the absence of any clear traumatic event, a condition known as atraumatic or spontaneous splenic rupture, which has a reported incidence of less than 1% [7]. ASR is a potentially life-threatening condition that affects males twice as much as females and occurs in middle-aged individuals [16]. While acute EBV infection is the most frequent cause of ASR, less common causes include cytomegalovirus infection, tuberculosis, melioidosis (caused by *Burkholderia pseudomallei*), amyloidosis, systemic lupus erythematosus, and splenic vein thrombosis associated with prothrombin gene mutations [16]. ASR can be categorized into seven types: neoplastic, infectious, hematological, inflammatory, iatrogenic, primary splenic, and idiopathic. Neoplastic and infectious conditions together account for more than half of all ASR cases [9].

Serious complications associated with EBV infections are often delayed. As EBV infection advances, mononuclear cells accumulate within lymphoid tissues, including the spleen. This leads to splenomegaly, and as the spleen enlarges, the splenic capsule becomes thinner, a condition seen in about 50% of patients. A secondary complication of this reversible splenomegaly is splenic rupture, as demonstrated in this case [1]. Splenic rupture is a rare but serious complication of EBV infection, occurring in 0.1-0.5% of patients, most commonly in males. It may present with diffuse, non-localized abdominal pain, nausea, or pleuritic chest pain. Palpation of the spleen can trigger left shoulder pain, referred to as Kehr’s sign [5]. A thorough systematic review of case reports on splenic rupture in IM from 1984 to 2014 found the average age of affected individuals to be 22 years [15]. CT is the preferred confirmatory test as it enables both the diagnosis and assessment of the severity of splenic injury. It is important to know that the absence of a history of trauma may delay the diagnosis of this potentially fatal complication, increasing mortality to approximately 30% [17].

According to a study by Toti et al. [18], 186 cases of splenic rupture had been documented between 1970 and 2023. Approximately 80% of cases occurred within three weeks of the onset of mononucleosis symptoms. The mortality rate of splenic rupture was 4.8%. Another study by Fugl and Andersen [4] estimated the incidence of splenic rupture in IM to be 0.1-0.2%. Another study by Busch et al. [2] reported that 50-60% of patients with EBV infection experience splenomegaly, with splenic rupture occurring in 0.5% of cases and a 30% mortality rate associated with it. Another study by Sylvester et al. [6] reported 42 cases of IM-induced ASR between 2006 and 2016. The average time to splenic injury was 15.4 (±13.5) days. Around 73.8% (n=31) of the injuries occurred within 21 days, while 90.5% (n=38) occurred within 31 days of symptom onset. In cases of minor splenic injury, conservative management with fluid resuscitation, with or without blood transfusions, along with close monitoring in an ICU, may be adequate. Splenectomy may be indicated when conservative management fails to achieve hemodynamic stabilization [7]. The American Association for the Surgery of Trauma (AAST) splenic injury grading scale is based on anatomical findings from CT scans and helps predict the likelihood of successful nonoperative management [1]. In cases in which observation or a trial of splenic embolization is not feasible, surgically mediated splenectomy is indicated. The current National Institute for Health and Care Excellence (NICE) guidance advises "to avoid contact or collision sports or heavy lifting for the first month of illness (to reduce the risk of splenic rupture)" [15].

## Conclusions

This case highlights the importance of the early recognition of splenic rupture as a possible complication of IM. Prompt diagnosis, early surgical intervention, and supportive care, including postoperative pain control, are essential for managing this life-threatening condition. Increased vigilance by healthcare providers in identifying risk factors and symptoms associated with splenic rupture can result in timely and appropriate management, leading to decreased morbidity and mortality.
